# RNA Analysis and Clinical Characterization of a Novel Splice Variant in the *NSD1* Gene Causing Familial Sotos Syndrome

**DOI:** 10.3389/fped.2022.827802

**Published:** 2022-02-02

**Authors:** Olatz Villate, Hiart Maortua, María-Isabel Tejada, Isabel Llano-Rivas

**Affiliations:** ^1^Pediatric Oncology Group, Biocruces Bizkaia Health Research Institute, Barakaldo, Spain; ^2^Neurodegenerative Diseases Group, Biocruces Bizkaia Health Research Institute, Barakaldo, Spain; ^3^Genetics Service, Hospital Universitario Cruces-Osakidetza, Barakaldo, Spain; ^4^Biocruces Bizkaia Health Research Institute, Barakaldo, Spain; ^5^Spanish Consortium for Research on Rare Diseases (CIBERER), Madrid, Spain

**Keywords:** next-generation sequencing, *NSD1*, Sotos syndrome, molecular analysis, RNA, splicing mutation

## Abstract

**Background:**

Sotos syndrome is an autosomal dominant disorder characterized by overgrowth, macrocephaly, distinctive facial features and learning disabilities. Haploinsufficiency of the nuclear receptor SET domain-containing protein 1 (*NSD1*) gene located on chromosome 5q35 is the major cause of the syndrome. This syndrome shares characteristics with other overgrowth syndromes, which can complicate the differential diagnosis.

**Methods:**

Genomic DNA was extracted from peripheral blood samples of members of the same family and targeted exome analysis was performed. *In silico* study of the variant found by next-generation sequencing was used to predict disruption/creation of splice sites and the identification of potential cryptic splice sites. RNA was extracted from peripheral blood samples of patients and functional analyses were performed to confirm the pathogenicity.

**Results:**

We found a novel c.6463 + 5G>A heterozygous *NSD1* gene pathogenic variant in a son and his father. Molecular analyses revealed that part of the intron 22 of *NSD1* is retained due to the destruction of the splicing donor site, causing the appearance of a premature stop codon in the NSD1 protein.

**Conclusions:**

Our findings underline the importance of performing RNA functional assays in order to determine the clinical significance of intronic variants, and contribute to the genetic counseling and clinical management of patients and their relatives. Our work also highlights the relevance of using *in silico* prediction tools to detect a potential alteration in the splicing process.

## Introduction

Sotos syndrome (OMIM #117550) is a rare autosomal dominant disorder characterized by overgrowth (increased height, macrosomia, and macrocephaly), characteristic facial features and learning and intellectual disabilities (1). Other associated clinical features include scoliosis, seizures, renal and cardiac anomalies (2, 3).

Sotos Syndrome is caused by heterozygous pathogenic variants in the nuclear receptor SET domain-containing protein 1 (*NSD1*) gene located on 5q35. *NSD1* encodes a histone methyltransferase that catalyzes the transfer of methyl groups to lysine residues of histone tails (4). The overall prevalence of this syndrome is estimated at 1 in 14,000 (5). More than 95% of the cases arise from *de novo* mutations and familial cases of Sotos syndrome account for about 5% of the diagnoses (6–8).

This syndrome shares characteristics with other overgrowth syndromes, which can complicate the differential diagnosis. The greatest phenotypic overlap is between Sotos and Weaver syndromes (5, 6). The clinical features of post-natal overgrowth, advanced bone age, and intellectual disability particularly in the context of mild facial dysmorphism can make it difficult to distinguish between these syndromes (6).

Targeted exome sequencing has become a powerful and useful method to diagnose patients with suspected overgrowth disorders (9). After performing next generation sequencing (NGS) studies, different novel variants of uncertain significance (VUS) can be detected in the studied patients so it is crucial to characterize their biological impact and determine their pathogenicity. In the case of variants that have been shown by bioinformatics programs to alter the splicing process, functional approaches such as RT-PCR analysis of patient-derived RNA or minigene splicing assays can be used to assess the effect of these variants on mRNA splicing (10, 11). The relevance of reclassification of splice variants classified as VUS lies in the fact that the patient needs to follow precise medical management, in accordance with the international guidelines (12, 13).

In this study, we report one novel intronic *NSD1* variant in two members of the same family. Clinical, familial and molecular data, together with our experimental RNA functional assays, have provided a pathogenicity characterization of the *NSD1* variant.

## Materials and Methods

### Patients

A Spanish boy and his father were recruited. This study was conducted in full accordance with the World Medical Association Declaration of Helsinki (version 2008) and the additional requirements. Informed consent approved by the clinical ethical committee of Cruces University Hospital (Spain) was obtained prior to genetic testing.

### Next Generation Sequencing Studies and Sanger Validation

Genomic DNA was extracted from peripheral blood and targeted exome analysis was performed in the Institute of Genomic Medicine (IMEGEN, Valencia, Spain). The analysis of the genes associated with overgrowth syndromes included: *AKT1, APC2, CDC45, CDC6, CDKN1C, CDT1, DICER1, DIS3L2, DNMT3A, EED, EZH2, FBN1, GMNN, GPC3, GPC4, H19, HERC1, IGF2, MCM5, MYO5A, NFIX, NSD1, OFD1, ORC1, ORC4, ORC6, PIK3CA, PTEN, RNF135, SETD2* and *SUZ12*. For this purpose, the exonic regions of interest were captured using the kit xGen Exome Panel v1.0 (IDT) and pair-end libraries were generated. Ultrasequencing was performed on the NextSeq 500 sequencing system platform (Illumina). Bioinformatics analysis of the data was done using Data Genomics Exon Pipeline software (version v1). Subsequently, the results were interpreted and prioritized. Changes with a number of readings >20 and with a frequency greater than 30% were considered variants. The variants detected were analyzed in four different databases (Exome Aggregation Consortium, 1000 Genomes, Human Gene Mutation Database and Exome Variant Server) and *in silico* prediction programs (SIFT, Polyphen-2, and MutationTaster-2). Conventional Sanger sequencing in genomic DNA validated candidate variant. Primers used were NSD1gF 5′-ACCCATTGCCACGGAAGAAA-3′ and NSD1gR 5′-CACCGCTGTCCCATTCTTCA-3′.

### Splicing *in silico* Analyses

*In silico* study of the variant found by NGS was used to predict disruption/creation of splice sites and the identification of potential cryptic splice sites. The following online programs were used: NNsplice (http://www.fruitfly.org/seq_tools/splice.html) and Human Splicing Finder (http://www.umd.be/HSF/).

### RT-PCR and cDNA Analysis

cDNA was obtained using Superscript RT II enzyme (Invitrogen, Carlsbad, CA, USA) from 500 ng of total RNA extracted from peripheral blood in a volume of 20 μl. cDNA was amplified and sequenced to identify potential splicing variants. Primers used for cDNA amplification and identification of splice variants were: NSD1cF 5′-CCAAAGAATCAACCCATTGC-3′ and NSD1cR 5′-GATTGCTCTGCCAGGTGAGT-3′. Primers for new splice variant sequencing were: NSD1svF 5′-AGCAGGTTGATGCCAAAATC-3′ and NSD1svR 5′-CTCTGCCAGGTGAGTGCTT-3′.

## Results

### Patients

**Figure 1:**
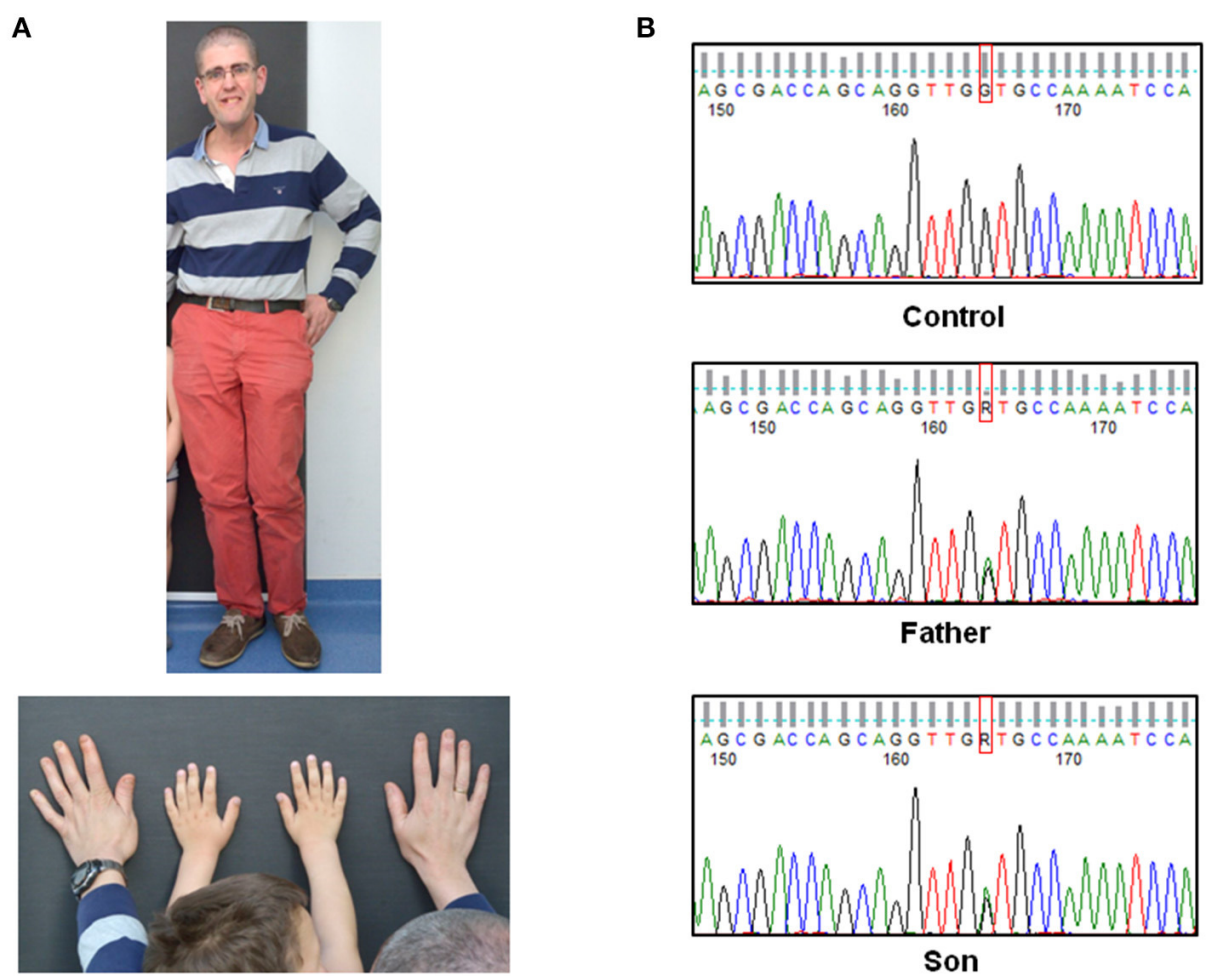
Physical characteristics of the patients and confirmation of the *NSD1* gene variant. **(A)** Photographs of the two patients showing large hands in both of them and macrocephaly, facial asymmetry and tall height in the father. **(B)** Electropherogram of the genomic sequence obtained by Sanger sequencing confirming the *NSD1* variant identified by targeted exome sequencing in the patients. The control did not present the variant.

The proband was a 3-year-old male patient being the second child of a non-consanguineous Spanish couple. He presented with plagiocephaly, asymmetric facies, high palate with decreased posterior palatal movement and uvula slightly deviated to the left. Prominent chin and prominent forehead were also present. Ear pinnae were well implanted. Chest and abdomen did not show alterations and no scoliosis was observed. Size was 111.5 cm (>p99 +3.3 SDS), height 23 kg (p99, +2.3 SDS) and head circumference 54 cm (>p99, +2.5 DS). Full limbs, symmetrical, with large hands and feet and thumbs of the four extremities wide (Figure 1A). Smooth skin, no spots, no asymmetric overgrowths and no alterations of subcutaneous tissue were noticed. Psychomotor development considered within normality except for delayed speech, language articulation problems, with communicative intention. No seizures. Features were shared with his 43-year-old father presenting with tall stature (184 cm, p84 +1.02 SDS), macrocephaly, facial asymmetry (Figure 1A) and velopalatine insufficiency. Intelligence was normal-low and he had no seizures. Paternal grandfather, deceased, seemed to present with macrocephaly and facial features similar to the father.

### Targeted Exome Analysis

Father's DNA was used for Next Generation Sequencing studies. In total 94.8% of the region of interest (coding region of the selected genes) was covered at least 20X. A heterozygous change was detected in intron 22 of the *NSD1* gene c.6463 + 5G>A (NM_022455.4). This change was not previously described and it was considered a variant of uncertain significance. In total 100% of the region of interest of the *NSD1* gene was covered with a depth greater than 20X. The coding region of this gene was covered with an average depth of 93.4X. The presence of this variant was validated in the father and his son by Sanger Sequencing (Figure 1B) and was not identified in the healthy sister of the proband.

### *In silico* Analyses

**Figure 2:**
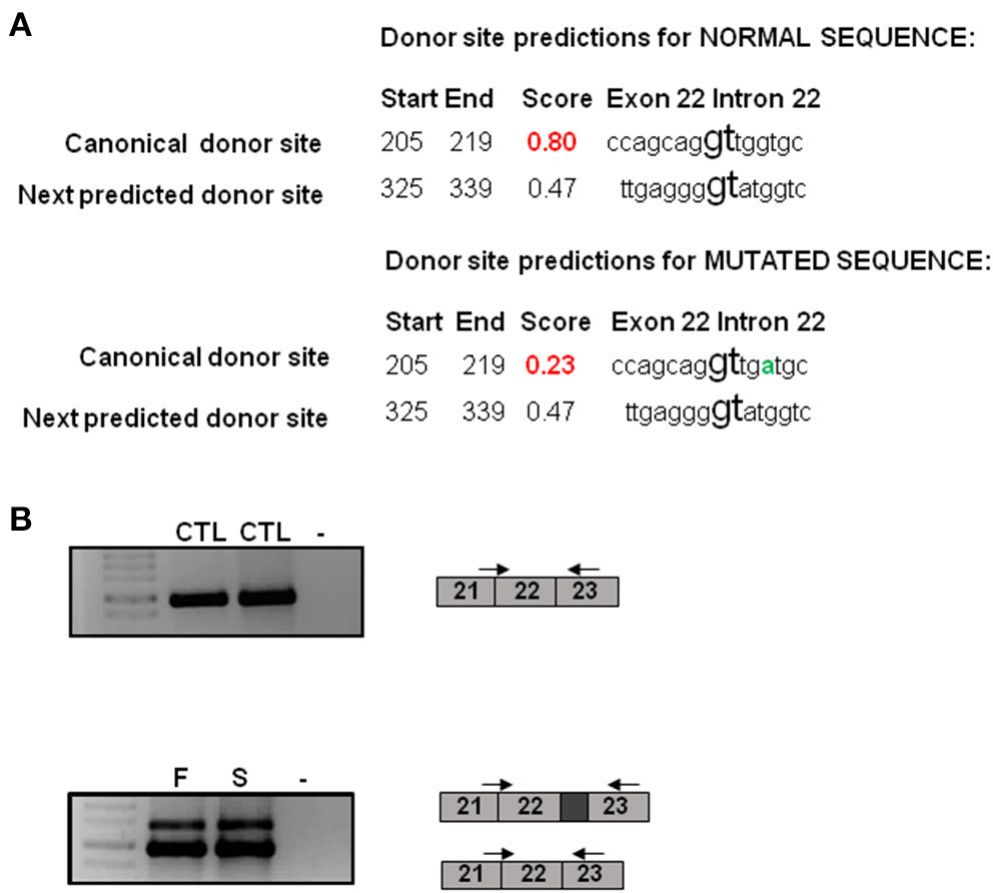
Molecular characterization of the *NSD1* variant. **(A)** Bioinformatics analysis of the c.6463 + 5G>A variant. NNsplice program predicts that the score for the donor splice site changes from a high value of 0.80 in the wild type sequence to a low value of 0.23 in the mutated sequence. The next donor splice site in the mutated sequence in the intron 22 is predicted to be 120 bp far from the first one. The variant change is represented in green. **(B)** RT-PCR analysis reveals an additional band in the father and the son in comparison to controls. This band is 120 bp longer than the other one. CTL, control; F, father; S, son.

The Human Splicing Finder program predicted an alteration of the donor site indicating a probable disruption of splicing in intron 22. The NNsplice program predicted that the score for the donor splice site changes from a high value of 0.80 in the wild type sequence to a low value of 0.23 in the mutated sequence indicating that the donor consensus sequence is weakened. The next donor splice site in the mutated sequence in the intron 22 was predicted to be 120 bp far from the first one indicating that the variant could induce the retention of part of the intron (Figure 2A).

### Molecular Characterization of the New Variant

**Figure 3:**
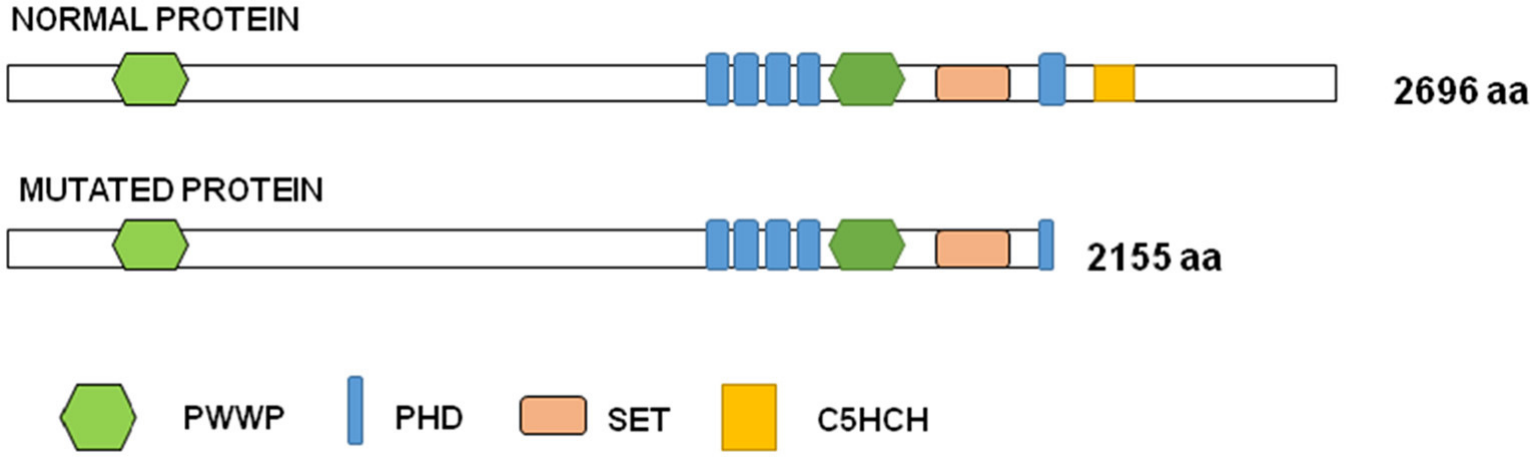
Graph representation of NSD1 protein. Functional domains are represented in colors in the normal and in the mutated proteins: PWWP (proline-tryptophan-proline-tryptophan) is represented in green, PHD (plant homeodomain finger) in blue, SET (SU[VAR]3–9,E[Z], trithorax) in orange and C5HCH (Cys-His-rich domain) in yellow.

To perform the functional studies, RNA samples were obtained from the father, the son and two healthy controls. *NSD1* transcripts were first analyzed by PCR in two controls. Only one band corresponding to the size of the normal transcript was observed and it was confirmed by sequencing (Figure 2B). Subsequently, the same study was performed on cDNA obtained from RNA from the father and the son and the results showed two bands, the band corresponding to the normal transcript and an additional one 120 bp longer than the normal one (Figure 2B). The two bands obtained in each case were sequenced. Sequencing confirmed that 120 bp of the intron were retained as predicted by *in silico* analysis. The retention of 120 bp has a potential impact on the protein as a premature stop codon is produced and a PHD finger and a C5HCH domain are affected in the mutated protein (Figure 3). This alteration confirms the pathogenicity of the variant.

## Discussion

In this study, we have combined molecular, clinical and functional analysis of two patients of the same family suspected of having an overgrowth syndrome. Our main aim was to contribute to the molecular and clinical classification of the variant of uncertain significance detected in *NSD1*.

The variant found in *NSD1* by targeted exome sequencing was mapped in the position +5 of the intron 22. It is well known that the GT dinucleotide at the 5′ end of an intron (positions + 1 and + 2) is highly conserved in human genes (> 98%) and critical for RNA splicing (14). These alterations are nearly systematically classified as pathogenic or probably pathogenic. In the position +5 of the 5′ splice sites the guanine is the most frequently nucleotide found, reaching 78% conservation among over 180,000 human 5′ splice sites (14) and it has been shown that disease-associated alterations are very often detected at this nucleotide (15). The variants at this position are usually classified as VUS so functional studies are required to determine their pathogenicity. Our results support the relevant role of intronic position + 5 in normal splicing and agree with previous studies showing that this position seems particularly prone to aberrant splicing when altered (10, 16). Moreover, we observed a good concordance between *in silico* programs used to predict splicing alterations and the results of the molecular assays for this variant located at the + 5 position.

To identify more disease causing variants in overgrowth syndromes there is a need to extend the studies to intronic variants. Due to the size of intronic regions, identifying deep intronic variants that affect splicing is challenging. The recent applications of whole-genome sequencing (WGS) to clinical screening studies enable the investigation of non-coding variation and identification of pathogenic deep intronic variants that lie >100 bp away from the nearest canonical splice sites (17).

In the Human Gene Mutation Database Professional 2020.4, 564 mutations have been reported in *NSD1*, 461 of them associated to Sotos Syndrome. The c.6463 + 5G>A variant is not reported in HGMD and our patients are the first ones described with this change. It is worthy to note that 24 splice variants are described as responsible for Sotos Syndrome phenotype and only one of them is mapped in position +5 of the splice site (18). This could suggest that functional studies are not being performed on variants at positions other than +1 and +2, which are necessary to confirm the diagnosis and provide correct genetic counseling. Of the 24 splice variants described in HGMD, 21 of them affect positions +1, +2, −1 and −2. The Human Splicing Finder program predicts in these variants an alteration of the donor or acceptor site depending on the position, indicating that most probably affect the splicing process. The variant previously described at position +5, c.4378 + 5G>C, according to bioinformatics analysis causes an alteration of the wild type donor site, most probably affecting splicing. There is also a splice variant described at position −5, c.6152-5T>G, and it is predicted to alter the wild type acceptor site, most probably affecting splicing. The last variant described that is not in the canonical positions is found at position +33, c.3796 + 33A>T, and in this case, the Human Splicing Finder prediction considers no significant impact on splicing signals. The NSD family members, consisting of NSD1, NSD2 and NSD3 are methyltransferases implicated in multiple diseases (19). We have shown that the variant found in *NSD1* has a potential impact on the protein causing a shortening and affecting C5HCH and PHD finger domains due to the appearance of a premature stop codon. NSD1 binds upstream of the bone morphogenetic protein 4 promoter, enforces H3K36 methylation levels within this region, and thus promotes bone morphogenetic protein 4 transcription (20). It has been shown that the PHD5-C5HCH domains of the NSD1 protein might have chromatin targeting ability and that Sotos syndrome mutations within these domains seem to alter the normal function of NSD1 (19). This would confirm the pathogenicity of the variant present in the two patients.

## Conclusions

Our findings underline the importance of performing RNA splicing assays in order to determine the clinical significance of intronic variants, and contribute to the genetic counseling and clinical management of patients and their relatives. Our work also contributes to highlight the relevance of using *in silico* prediction tools to detect a potential alteration in the splicing process.

## Data Availability Statement

The original contributions presented in the study are included in the article/supplementary materials, further inquiries can be directed to the corresponding author/s.

## Ethics Statement

The studies involving human participants were reviewed and approved by Clinical Ethical Committee of Cruces University Hospital (Spain). Written informed consent to participate in this study was provided by the participants' legal guardian/next of kin. Written informed consent was obtained from the individual(s), and minor(s)' legal guardian/next of kin, for the publication of any potentially identifiable images or data included in this article.

## Author Contributions

OV and HM performed the bioinformatics and molecular analyses. M-IT directed the project granted and reviewed the manuscript. IL-R carried out the clinical characterization of patients. OV and IL-R conceived the study, supervised it, and wrote the manuscript. All authors have read and approved the final manuscript.

## Funding

This work was supported by grant CIBERER17-TRASL7-02 from CIBERER (Spanish Consortium for Research on Rare Diseases).

## Conflict of Interest

The authors declare that the research was conducted in the absence of any commercial or financial relationships that could be construed as a potential conflict of interest.

## Publisher's Note

All claims expressed in this article are solely those of the authors and do not necessarily represent those of their affiliated organizations, or those of the publisher, the editors and the reviewers. Any product that may be evaluated in this article, or claim that may be made by its manufacturer, is not guaranteed or endorsed by the publisher.
